# Are Older Adults a Burden on Society? Ethnic Differences in What Younger Nigerians Think

**DOI:** 10.1093/geroni/igaa057.1036

**Published:** 2020-12-16

**Authors:** Abolade Oladimeji

**Affiliations:** case western reserve university, cleveland, Ohio, United States

## Abstract

Ageism is on the rise in Nigeria. Recently congress passed a bill on “not too young to rule” a campaign to advocate for younger Nigerians to take over leadership positions from older political officeholders. This paper focuses on ethnic differences among Nigerians who think older adults are a burden on society. Our sample included 1,693 randomly selected Nigerians (18 yrs-59yrs) who were in wave six of the world values survey conducted in 2012 comprising of the three major ethnic groups in Nigeria; Hausa (33%), Igbo (24%), and Yoruba (25%). Using multivariate logistic regression, results show that among the Hausas; life satisfaction, education, employment, gender, income, and geography predict bias towards older adults (OR=1.12*,.89*,.61*,.63*,1.12*,.74***) respectively. Among the Igbos; only education predicts bias towards older adults (OR=.75***). Among the Yorubas; income and geography predict bias towards older adults (OR=.86*, 1.20*) respectively. These findings underscore that several individual resources, as well as relationship status, are associated with a bias towards the elderly in Nigeria. The results reinforce the idea that the underlying causes of ageism might be structural such as political failure, poverty, illiteracy, and rurality. To change how younger generations, think of older adults, macro-level interventions and policies need to be implemented.

